# Supplementary material for: Are carbon emissions trading and green financial instruments synergistic? -Comprehensive quantitative research based on content analysis
Source: PLoS One. 2024 Mar 7;19(3):e0298601. doi: 10.1371/journal.pone.0298601 (PMC10919601; doi:10.1371/journal.pone.0298601)
Supplement: S1 Data — (ZIP) [file pone.0298601.s001.zip › Data/地级市Z_F工作报告.zip!/║╙▒▒/└╚╖╗╩╨/└┤╫╘╣1⁄2╓┌║┼í╛┬φ┐╦╩2╛▌═°í┐_2016.docx]

各位代表：

从今年开始，我们已进入第十三个五年规划时期。根据党的十八届五中全会、省委八届十二次全会和市委五届八次全会精神，市政府编制了《廊坊市国民经济和社会发展第十三个五年规划纲要(草案)》。现在，我代表廊坊市人民政府向大会报告工作，请各位代表连同《纲要(草案)》一并审议，并请各位政协委员和列席人员提出意见。

一、“十二五”时期经济社会发展回顾

“十二五”时期是廊坊应对严峻复杂形势、综合实力持续增强的五年，是产业结构转型升级、发展方式加快转变的五年，是社会事业全面进步、群众得到更多实惠的五年。特别是党的十八大以来，在省委、省政府和市委坚强领导下，全市上下深入学习贯彻习近平总书记系列重要讲话精神，主动适应、积极引领经济发展新常态，顽强拼搏、砥砺奋进，胜利完成“十二五”规划主要目标任务，在全面建成小康社会进程中迈出坚实步伐。

这五年，我们积极应对经济下行压力，着力扩内需稳增长，综合实力跨上新台阶。地区生产总值从2010年的1351亿元增至2015年的2401.9亿元，年均增长9.4%，人均地区生产总值由3.2万元增至5.3万元，年均增长8%;全部财政收入从195.4亿元增至481.3亿元，年均增长19.8%;一般公共预算收入从105.9亿元增至303.4亿元，年均增长23.4%，成为全省第三个突破300亿元的设区市;全市人均财力由3313元提升到8133元;全社会固定资产投资从909亿元增至2160亿元，年均增长18.9%;规模以上工业增加值从451.5亿元增至721.6亿元，年均增长9.5%;社会消费品零售总额从419.1亿元增至793.2亿元，年均增长13.5%。经济发展的质量效益持续提升，全部财政收入占地区生产总值比重由14.5%提高到20%，一般公共预算收入占地区生产总值比重由7.8%提高到12.6%，民营经济上缴税金占全部财政收入比重由84%提高到89%。全部财政收入总量由全省第六位升至第三位。一般公共预算收入总量由全省第四位升至第三位，占全省比重由9.2%提高到11.5%。全市综合实力实现大幅提升。

这五年，我们抢抓京津冀协同发展重大机遇，主动作为、深度对接、重点突破，协同发展实现良好开局。积极落实《京津冀协同发展规划纲要》，建立推进机构，制定实施意见，明确了“三区一地”发展定位。持续强化与京津常态化沟通，扎实推进交通、产业、生态等领域务实合作，与大兴、通州、西城、武清等地签订战略合作协议。立体化交通网络加速形成。北京新机场全面开工，新机场外围“五纵两横”综合交通规划获批，廊沧、京台高速廊坊段和京沪、津保高铁建成通车，京廊通勤高铁开通运行，京唐、廊涿、京霸、平谷线、白沟支线等轨道交通项目顺利推进，京秦、密涿高速等断头路加速对接，燕郊潮白河大桥加快建设。产业协同持续深化。中兴研究院、人保北方信息中心、京东华北订单处理中心、微软游戏创新中心等一批企业区域总部落户，中轻造纸装备、联通华北基地等一批重大项目投产，鲁能生态度假区、国电热电联产等一批央企项目开工，固安航天产业基地、中集空港专用装备等一批基地型项目加快推进。功能承接实现突破。燕达医院与北京朝阳、天坛医院共建共管，率先建立医保异地就医结算机制，北京五中、八中等一批学校分校落户，京廊医疗、教育、文化、养老等合作深度拓展。生态共建不断强化。区域生态环境信息共享、大气污染联防联控联治取得成效。协同发展为廊坊带来的全方位、历史性变化正在日益显现。

这五年，我们大力推进结构调整，积极构建绿色循环低碳现代产业体系，转型升级取得重大进展。项目建设取得扎实成效。坚持以增量优化带动存量调整，建设亿元以上项目2289项，高端制造、现代服务业项目占比达到60%以上，争列省重点项目199项，居全省首位。航天振邦、智能机器人产业港、精雕数控机床等一大批高端项目建成投产。三次产业比例由11.6:53.6:34.8调整优化为8.6:44.3:47.1，产业结构呈现“三、二、一”格局，实现历史性突破。新兴产业支撑作用不断增强。电子信息产业增加值年均增长16.8%，成为发展最快的支柱产业，占规上高新技术产业增加值比重达到44.3%;高端制造业增加值从107.4亿元增至201.8亿元，年均增长13.4%，占规上工业增加值比重达到28.4%;服务业增加值从470亿元增至1131.3亿元，总量超过工业，对经济增长的贡献率从38.1%提高到61.6%。传统产业改造步伐持续加快。圆满完成压减钢铁、水泥、平板玻璃等过剩产能任务，单位GDP能耗提前一年完成省定任务。累计实施工业技改项目3281项，投入工业技改资金2334亿元，近两年工业技改投资年均增长24.1%，增幅居全省前列。近三年全市新增规上工业企业460家。现代农业蓬勃发展。建成省级农业科技园区11家、省级现代农业园区4家，大厂、固安获批国家级农业科技园区，6个县(市、区)入围环首都现代农业科技示范带，永清县入选国家级现代农业示范区，省级以上农业产业化龙头企业达到48家、国家级5家，农业产业化经营率达到67.5%。

这五年，我们深入实施创新驱动发展战略，全力打造科技成果孵化转化引领区，创新能力大幅提升。规上高新技术产业增加值达到170亿元，五年增长1.16倍，占规上工业增加值比重达到23.9%，五年提高6.4个百分点。创新主体持续增加。科技型中小企业数量从2013年的1211家增至3262家、科技小巨人企业从2014年的59家增至171家，均居全省前列。创新平台建设全面加快。建成国家级高新区1家、省级高新区5家、省级以上研发机构43家、院士工作站14个，建设了中科院廊坊战略性新兴产业孵化基地、清华大学中试孵化基地、北大科技成果展示交易中心、北航机器人研究所等一批协同创新平台。创新环境持续优化。出台系列扶持政策，引进国家“千人计划”专家14人，设立10支、17亿元科技金融基金，科技进步贡献率达到48%，专利授权量9082件，是“十一五”的3.2倍。我市被评为全国科技进步考核先进市。

这五年，我们以壮士断腕的决心，强力推进生态环境治理，环境质量持续改善。坚决向大气污染宣战。实施科学治霾、依法治污，近三年财政投入大气污染防治资金23.6亿元。深入开展“蓝天行动”，强力实施控煤、抑尘、限车、治污、治烟等重点工程，全部取缔实心粘土砖瓦窑，全部淘汰现有黄标车，取缔非法排污企业1329家，全面完成“十二五”主要污染物总量减排任务。PM10、PM2.5平均浓度比2013年分别下降25.5%和22.7%，为京津冀区域空气质量改善、营造“APEC蓝”和“阅兵蓝”做出了重要贡献。认真落实《环境保护法》，深入实施“利剑斩污”专项行动，严厉打击环境违法犯罪，有力震慑了环境违法行为。强力推进水环境治理。对重点河流开展全流域综合治理，新建和提标改造污水处理厂7座，流域水质不断改善。完成京南森林湿地生态走廊项目论证，潮白河大运河湿地公园列入国家级湿地公园试点，赵王新河湿地公园成为省级湿地公园。大力推进植树增绿。实施了“两年攻坚战、造林一百万”等工程，五年累计造林164万亩，大厂成为全国绿化模范县，三河、香河通过全国绿化模范县验收。

这五年，我们全面深化改革开放，着力破解体制机制障碍，发展动力活力不断增强。成功争列第一批全国中小城市综合改革试点，列入国家全面创新改革试验区域。简政放权深入推进。全部取消非行政许可审批事项，市级行政审批事项五年削减29.7%，在全省率先实现投资项目在线审批，廊坊开发区率先开展综合改革，成立行政审批局。“一办两中心”、“三级平台”全面建成，“两个代办”高效运转，“三个清单”编制发布，市县政府机构改革全面完成，挂牌成立市县不动产登记局和登记中心，食品药品监管、国资等改革取得扎实成效，固安PPP合作模式获得国务院办公厅通报表扬。商事制度改革实现突破。全面实行“三证合一”、“一照一码”，五年新增市场主体15万户，万人拥有市场主体由改革前的398家提高到487家，四个县(市)跻身全省民营经济十强县。财税金融改革全面推进。绩效预算改革全面推开，“营改增”深入实施，引进7家域外银行，存贷款余额均比2010年增长1.5倍，新增挂牌上市企业39家。农村综合改革取得积极进展。农村土地确权登记颁证全面展开，土地经营权流转85.7万亩。对外开放广度深度不断拓展。世界500强企业达到29家，实际利用外资33.8亿美元，比“十一五”增长64.5%，累计外贸出口134.3亿美元，比“十一五”增长59.5%。“5·18”、“9·26”展洽会知名度和影响力不断提升，成功举办军民融合展洽会、绿色产业博览会等系列活动，廊坊成为中国十佳优秀会展城市。省级以上开发区达到39家，居全省第一位，聚集了80%的新项目，财政收入超10亿元的开发区达到6个。开发区成为对外开放的战略平台，为又好又快发展注入了强劲动力。

这五年，我们统筹城乡发展，加快新型城镇化进程，城乡面貌发生较大变化。城镇布局全面优化。完成城市总规修编和城乡统筹规划、各县(市)城乡总规编制，中心城市确立“大四点”、“小四点”组团发展格局，全市常住人口城镇化率达到54%，五年提高5.2个百分点。中心城市加快发展。累计投入34.5亿元，新改扩建艺术大道、银河路、自然公园、京沪高铁站等一批基础设施和公共服务设施，建设了万达广场、浙商广场、红星美凯龙等一批城市综合体，在全省率先实施市、县两级数字城管，被列为国家智慧城市试点。县域经济实力全面增强。集中开展了县城和重点镇提升行动，建设了一大批科教文卫、路水气热、垃圾处理等基础设施，打造了一批特色街区和风貌建筑，综合承载能力不断提升。所有县(市、区)全部财政收入均超过10亿元，其中8个超30亿元、4个超50亿元。三河、固安、香河一般公共预算收入总量进入全省十强。基础设施全面加强。公路总里程突破1万公里，路网密度达到160公里/百平方公里，高速公路通车里程达到341公里，新建农村公路1249公里。南水北调三大干渠全面建成，廊涿干渠和天津干渠正式供水，8座地表水厂全部竣工，在全省率先具备受水区全域通水条件，引调黄河水2.6亿立方米，建成文安县城防洪圈。新建110千伏及以上变电站26座，移动通信4G网络实现全覆盖。农村人居环境持续改善。高标准建设省级新民居示范村392个，重点打造了北三县和固安两大美丽乡村省级重点片区和6个市级精品示范片，851个村街村容村貌显著提升，全省美丽乡村建设现场观摩会议连续两年在廊坊召开。

这五年，我们坚持发展成果人民共享，全面保障和改善民生，社会大局保持和谐稳定。民生投入力度持续加大。加快公共资源向基层延伸、向农村覆盖、向困难群体倾斜，持续实施就业、社保、教育、卫生、安居、帮困等民生工程，财政民生支出累计达到1239.9亿元，是“十一五”的3倍，占一般公共预算支出比重达到81.2%，比2010年提高了7.6个百分点。居民收入持续增长。城乡居民人均可支配收入比2010年分别增长62.5%和84.2%，城镇累计新增就业25.8万人，城镇登记失业率连续十年全省最低，零就业家庭动态归零。社会保障体系不断完善。城镇、农村低保标准全省最高，比2010年分别提高76.2%和146.8%，五保集中供养率保持全省领先。新农合和城镇居民医保财政补助标准均比2010年增长2.2倍，新农合参合率保持在95%以上，城镇基本医疗保险实现市级统筹，全面推行城镇职工和新农合大病商业保险，在全省率先实施重特大疾病医疗救助制度，全面实现新生儿35种遗传病免费筛查。城镇企业退休人员基本养老金连续11年上调，机关事业单位养老保险制度改革启动实施，城乡居民养老保险参保率保持全省领先，市区社区居家养老服务设施覆盖率达到93%。惠民实事扎实开展。开工建设保障性安居工程7.5万套，城镇低收入家庭住房保障应保尽保;改善老旧小区80个，7万多市民受益;建设农村集中供水水厂138座，在全省率先基本解决农村饮水安全问题;投资5.56亿元，购置公交车513辆，新增公交线路15条，市区公交出行分担率从5.7%上升至13%。社会事业全面进步。各级各类教育长足发展，促进教育公平取得新进展，投资30.8亿元实施农村校舍安全工程，576所学校竣工，30余万师生搬入新校舍。医药卫生体制改革成效明显，县级公立医院综合改革深入推进，公共卫生和医疗服务体系覆盖城乡。公共文化服务体系不断完善，市群艺馆、县乡村“两馆一站一室”达标建设进展顺利，荣获“中国书法城”、“中国合唱基地”，举办幸福廊坊文化艺术节、半程马拉松、第什里风筝节等一批文体活动。全民健身条件大幅改善，体育健身设施覆盖70%村街和85%以上社区。法治廊坊、平安廊坊建设扎实推进，社会治安综合治理能力不断提升，安全生产形势持续好转，食药安全监管力度不断加大，信访积案化解工作取得扎实成效。国防动员、双拥共建深入开展。香河县荣获全国综治工作最高奖“长安杯”。大厂县荣获全国民族团结进步模范集体荣誉。民族宗教、新闻出版广电、外事侨务、人民防空、史志档案、气象地震、地理信息、援藏援疆、妇女儿童、老龄、残疾人等各项工作都取得了新成就。

这五年，我们积极转变政府职能，驰而不息改进作风，政府自身建设明显加强。以开展党的群众路线教育实践活动、“三严三实”专题教育为契机，严格落实中央“八项规定”，加大正风肃纪力度，全面清理吃空饷人员，规范公务用车、办公用房和公务接待，“四风”问题得到有效遏制。深入推进依法行政，坚持依法科学民主决策，不断加强制度建设，严格规范行政执法行为，有效化解行政争议，法治政府建设迈出重要步伐，成为全国完善规范性文件合法性审查试点单位。积极推进党风廉政建设，认真落实党内两项法规，持续强化行政监察和审计监督，政府公信力和执行力进一步提高。开通961890群众服务热线，解决了一大批群众反映的困难和问题。自觉接受人大法律监督、政协民主监督和社会舆论监督，建立向人大报告和向政协通报工作的常态机制，承办人大代表建议和政协提案2353件，全部及时办复。

刚刚过去的2015年，我们坚持解放思想、抢抓机遇、奋发作为、协同发展，树正气、讲团结、聚合力、促转型，经济总量大幅提高、发展速度领跑全省、结构实现质的突破。地区生产总值同比增长8.8%，全部财政收入增长18.3%，一般公共预算收入增长21.1%，全社会固定资产投资增长16%，规模以上工业增加值增长6.1%，服务业增加值增长13.8%，社会消费品零售总额增长9.6%，实际利用外资增长5.4%，存贷款余额分别增长27.4%和30.6%。地区生产总值、全部财政收入、一般公共预算收入、全社会固定资产投资、服务业增加值、存贷款余额等指标增速居全省第一位，规模以上工业增加值、社会消费品零售总额指标增速居全省第二位。一年来，我们突出在六个方面下功夫。一是着力保运行、稳增长。积极应对严峻经济形势，找准着力点、狠抓增长点，全力以赴强化调度运行，年初制定了必保完成和争取完成两套指标，调高了标尺、激发了干劲;实施“十项集中行动”，迅速推进各项部署，全年工作强势开局;建立每月两次分析调度机制，定期研判财政入库，全力保障经济运行;扭住重点项目集中攻坚，实施一线推动和强力督导，确保如期开工;大力开展企业帮扶，面对面解决实际困难，全力帮助企业发展，经济实现逆势上扬。二是着力谋合作、促协同。加大力度推进与京津全面对接，办成了一批多年想办而没有办成的大事要事。共建园区实现突破，北京新机场临空经济区建设扎实推进，北京亦庄·永清高新区获批省级高新区，机构设置获省批复，被列为省“十三五”重大建设平台，首批7个项目开工建设;西城金融商贸产业园确定选址，永清国际商贸中心开工建设，京冀通航产业园、大兴固安电子商务产业园、中关村(大兴)生物医药产业基地固安合作园等签订合作协议。全面开展产业对接，京津冀产业转移系列对接活动成功签约项目37项，总投资1685亿元。三是着力增投资、上项目。强力实施项目攻坚十条和项目建设“十百百千”工程，建立了重大项目定期协调推进机制，建设亿元以上项目841项、完成投资1766.7亿元，列入省重点项目33项，居全省第一位，项目发展势头强劲;着力破解土地制约，闲置土地处置完成率达94%，违法占地处置土地到位率达99.9%。深入实施工业强市、服务业兴市战略，强力开展产业集群培育、工业基金引导、企业上市、技术对接、民企服务五大工程，助推产业转型发展。完成工业投资1057.3亿元，同比增长20%，其中工业技改投资716.1亿元，增长25%，新增规上工业企业137家，工业的支撑作用进一步凸显;实施现代服务业发展三年行动计划，强力推进八大专项重点突破，新增限上商贸企业66家;突出把大数据作为战略性新兴产业加以培育，成立大数据应用服务中心，润泽科技、华为云总部、联通华北基地等龙头企业的带动作用不断增强，与京东集团、浪潮集团等签订一批大数据产业合作项目，成功举办大数据产业周。四是着力抓创新、强动能。坚持把创新作为发展的第一动力，编制科技成果转化总体规划，大力实施“一十百千万”科技创新行动，新增科技型中小企业1500多家，是2014年的两倍多，新增科技小巨人企业112家;建成市级众创空间20家、省级2家，建成市级孵化器9家、省级11家，廊坊企业在美国、以色列及深圳、上海等地建成孵化器突破10家，孵化面积突破100万平方米，集聚科技企业和创客团队800多个;全国第一家博士后成果转化基地落户我市，与北京院所共建河北钢木家具产业技术研究院、绝热节能材料研究院。着力强化金融创新，建立京津廊金融家联盟，引入华夏银行、建信人寿等4家金融机构，发行公司债直接融资172亿元，居全省第一位，新增挂牌上市企业23家，其中3家在香港联交所上市。五是着力治污染、优环境。以更大的决心和力度，以最严格的措施和标准，强力推进大气、水污染治理。出台大气污染防治攻坚十条，高频率召开大气污染防治专题会议，实行市县主要领导周调度制度，成立多个督导组进厂入企下工地，网格化监管实现全覆盖，突出抓好重污染天气“削峰减频”，启动应急响应17次。全部淘汰市县建成区10蒸吨及以下燃煤锅炉，完成12.9万户农户清洁炉具替代，推广型煤48万吨、神华清洁煤151.7万吨，三河电厂完成超低排放改造。PM2.5同比下降15%，PM10下降13.8%，空气质量综合指数下降10.9%，达标天数增加32天，重污染天数减少19天，完成大气污染治理任务。积极开展重点河流水污染治理，流域水质得到改善。六是着力办实事、惠民生。持续加大民生投入，全力解决群众最关心、最直接、最现实的利益问题，教育、医疗、社保就业财政支出分别增长35.9%、27.1%和44.6%，新改扩建104所中小学、幼儿园，免除51.9万农村中小学生学杂费，资助2.6万名困难学生接受高中和中等职业教育;城乡居民人均可支配收入分别达到31900元和13100元，继续保持全省第一和第二位。我们以扎实的工作全面完成了2015年的各项任务，取得了丰硕的成果，实现了“十二五”圆满收官。

各位代表，在看到成绩的同时，我们也清醒地认识到存在的问题：经济发展内生动力不足，传统产业占比高，新兴产业支撑弱，转型升级任务艰巨;大气和水污染严重，土地制约日益加剧，资源环境压力比以往任何时候都更加突出;城市承载力亟待加强，基础设施、公共服务和城市治理存在不少短板;经济社会发展不够协调，民生欠账依然突出，全面建成小康社会仍有很多薄弱环节;营商环境有待提升，政府职能转变仍需加强，一些干部思想不够解放，适应引领新常态仍有大量工作要做。对此，我们一定高度重视，积极采取有效措施认真加以解决。

各位代表，回顾过去五年的奋斗历程，面对速度变化、结构优化、动力转换的新常态，面对国内外严峻复杂的新形势，面对治污染、调结构、防风险多重压力的新挑战，取得的成绩来之不易，积累的经验弥足珍贵。我们深刻体会到，推动经济社会又好又快发展，必须认识适应引领经济发展新常态，不折不扣地贯彻落实中央的重大决策和省委、省政府的部署要求，转变发展理念，强化质量效益意识，坚定不移走生态优先、绿色发展之路;必须抢抓用好协同发展重大机遇，顺势而为、借势发力，把主要精力、关键举措集中在协同发展上，在协同发展中增实力、优结构、补短板，真正把机遇优势转化为发展优势;必须坚持改革创新引领发展，以改革破难题，以创新增动力，积极抢占发展制高点，使改革创新成为推动发展的强大引擎;必须把转型升级作为主攻方向，坚持高端引领，加快转型步伐，构建特色鲜明的现代产业体系，全面提升发展层次和水平;必须优先加强生态建设，牢固树立绿水青山就是金山银山的理念，以生态约束倒逼发展转型，以发展转型保障生态改善，重塑环境优势，实现可持续发展;必须增进人民群众福祉，优先增加民生投入，不断提高公共产品供给的质量和效率，使发展成果更多更公平惠及全市人民。

各位代表，“十二五”目标任务的圆满完成，是我们认真贯彻党中央和省委、省政府正确决策的结果，是市委总揽全局、坚强领导的结果，是人大、政协加强监督和社会各界鼎力支持的结果，是全市人民齐心协力、奋力拼搏的结果。在此，我代表市人民政府，向全市人民，向人大代表、政协委员，向各民主党派、工商联、无党派人士和人民团体，向驻廊解放军、武警官兵、政法干警和中省直驻廊单位，向所有关心支持廊坊发展的同志们、朋友们，致以衷心的感谢和崇高的敬意!

二、“十三五”时期主要目标和任务

廊坊在全省发展大局中位置特殊、责任重大、作用重要。“十三五”是我市发展历史上重大机遇最为集中的时期，是各种优势潜力最能有效释放的时期，是破解难题补齐短板最为紧要的时期，是推进结构调整又好又快发展最为宝贵的时期。省委、省政府对廊坊发展寄予厚望，要求我们加压奋进，创造新经验、做出新示范，率先走出一条生态优先、绿色发展的新路，为全省发展多作贡献。全市人民热切期盼更加幸福的生活，强烈期待我们把廊坊建设得更加美好。使命考验担当，责任无比重大。

面对复杂多变的严峻形势，面对不进则退的竞争挑战，我们能否承担好历史和人民赋予的重大使命，能否在新一轮竞争中实现跨越争先，关键在干部队伍，关键在精神状态。只有锐意进取，才能克难前行，只有不断解放思想，才能开辟发展新路径。我们必须殚精极虑、激情工作，以无愧于时代的使命感、无悔于机遇的紧迫感、无畏于挑战的责任感，奋力开创廊坊发展新局面。

“十三五”时期我市经济社会发展的指导思想是：全面贯彻党的十八大和十八届三中、四中、五中全会及省委八届十二次全会精神，以邓小平理论、“三个代表”重要思想、科学发展观为指导，深入贯彻习近平总书记系列重要讲话精神，按照“五位一体”总体布局和“四个全面”战略布局，牢固树立创新、协调、绿色、开放、共享的发展理念，坚持“协同发展、转型升级、又好又快”工作主基调，以提高发展质量和效益为中心，坚守发展、生态、民生三条底线，牢牢抓住新机遇，努力适应新常态，按照市委五届八次全会的部署，大力实施协同发展、科技领引、开放带动、生态支撑主战略，着力做强城市经济，着力发展高端产业，着力创新体制机制，着力重塑环境优势，着力防范社会风险，加快建设科技研发创新成果转化引领区、战略性新兴产业和现代服务业聚集区、京津冀全面创新改革试验区、北京非首都功能集中承载地，努力率先走出一条生态优先、绿色发展的新路，奋力开创绿色、高端、率先、和谐发展新局面，确保提前全面建成小康社会，为加快建设经济强省、美丽河北作出新的更大贡献。

“十三五”时期经济社会发展的总体目标是：“三个前列、两个翻番、一个提前”。“三个前列”，就是经济增长保持中高速，主要经济指标增速位居全省前列，地区生产总值超过3900亿元，年均增长8.5%;产业发展迈入中高端，质量和效益位居全省前列，一般公共预算收入接近500亿元，年均增长10%以上;环境治理大见效，生态建设水平位居全省前列，PM2.5浓度下降25%。“两个翻番”，就是地区生产总值和城乡居民人均可支配收入比2010年翻一番以上，并努力在全省率先实现翻番。“一个提前”，就是确保在2020年前全面建成小康社会。

实现“十三五”发展目标，我们要始终坚持发展是硬道理的战略思想，变中求新、新中求进、进中突破，推动发展不断迈上新台阶;我们要始终坚持以发展新理念统领全局，真正做到崇尚创新、注重协调、倡导绿色、厚植开放、推进共享;我们要始终坚持认识适应引领经济发展新常态，自觉把思想和行动统一到习近平总书记提出的“十个更加注重”上来，确保中央和省委、省政府的决策部署落地生根;我们要始终坚持大力推进供给侧结构性改革，提高供给体系质量和效率，提高全要素生产率，着力解决制约发展的深层次问题;我们要始终坚持创新驱动发展战略，加快新动能成长和传统动能提升，努力走出一条生态优先、绿色发展的新路。

今后五年，我们将强力推动协同发展开创新局面。协同发展是实现生态优先、绿色发展的核心抓手。紧紧围绕“三区一地”发展定位，精准打造承载平台，构筑北中南三大协同发展功能区，构建对接京津区域新格局。到2020年，基本建成与京津内外连通、便捷高效的现代立体交通网络，承接北京非首都功能疏解和产业转移取得重大突破，“京津研发、廊坊转化”的产业协同模式和共建共享、联防联控的生态建设格局基本形成，经济社会发展和公共服务接近或达到京津平均水平。

今后五年，我们将强力推动科技领引取得新突破。科技创新是实现生态优先、绿色发展的第一动力。必须把创新摆在核心位置，更加注重提高发展质量和效益，强化政策激励、金融支持、人才保障和知识产权保护，扎实推进大众创业、万众创新，努力打造京津冀科技创新成果孵化转化基地。到2020年，全社会研发投入占GDP比重达到3%，科技创新能力明显提升，体制机制创新取得重大突破，初步建立起特色鲜明的区域协同创新体系，科技型中小企业达到7000家以上，科技进步贡献率达到60%。

今后五年，我们将强力推动开放带动迈出新步伐。改革开放是实现生态优先、绿色发展的必由之路。坚持问题导向，全面深化改革，努力在推进供给侧结构性改革上取得突破，主动融入国家对外开放大格局，加快构建开放型经济新体制。到2020年，重点领域、关键环节改革取得决定性成果，改革红利充分释放，双向开放水平显著提高。

今后五年，我们将强力推动生态支撑形成新优势。环境友好是实现生态优先、绿色发展的重要保障。坚守生态底线，实行最严格环境保护制度，强力治理大气污染，持续推进造林绿化，狠抓生态环境修复，高效节约利用资源，促进形成绿色生产方式和生活方式。到2020年，空气质量优良天数达到72%，重点河流水质达到国家考核指标要求，单位GDP能耗、主要污染物排放达到国家环境质量控制和总量控制要求，森林覆盖率稳定在30%以上。

今后五年，我们将强力推动转型发展实现新提升。转型升级是实现生态优先、绿色发展的关键所在。借力“中国制造2025”和“互联网+”行动计划，以供给侧结构性改革为重点，促进战略性新兴产业规模化发展，推动现代服务业做大做强，加快传统产业提质增效，推进现代农业做优做精。到2020年，产业结构进一步优化，现代产业体系基本形成，第三产业增加值比重提高到53%。

今后五年，我们将强力推动统筹发展构筑新格局。统筹城乡是实现生态优先、绿色发展的内生动力。推进以人为核心的新型城镇化，实施中小城市综合改革，推动城乡基础设施一体化、公共服务均等化和产业发展融合化。做大做强城市经济，壮大提升县域经济，全面推进美丽乡村建设。到2020年，将中心城市建设成京津冀世界级城市群中的重要节点城市，功能更加完备，承载能力显著提升。城镇体系更加完善，在全省率先实现美丽乡村全覆盖，全市常住人口城镇化率达到62%，户籍人口城镇化率达到45%。

今后五年，我们将强力推动共享发展达到新水平。共享发展是实现生态优先、绿色发展的根本目的。持续加大财政投入力度，着力抓好各项民生社会事业，不断健全社会治理体系。到2020年，城乡居民人均可支配收入分别达到4.8万元和2万元，各项社会事业全面进步，社会文明程度和治理水平进一步提升，人民幸福感、获得感明显增强。

各位代表!蓝图已经绘就，号角已经吹响。我们坚信，在省委、省政府和市委的坚强领导下，只要我们万众一心、奋力拼搏，就一定能够实现全面建成小康社会的宏伟目标，就一定能够建设一个更加繁荣、更加美丽、更加幸福、更加和谐的新廊坊!

三、2016年主要工作任务

今年是全面建成小康社会决胜阶段的开局之年，是京津冀协同发展的关键之年，是推进供给侧结构性改革的攻坚之年。我们要全面贯彻省委、省政府和市委的决策部署，按照宏观政策要稳、产业政策要准、微观政策要活、改革政策要实、社会政策要托底的要求，切实抓好去产能、去库存、去杠杆、降成本、补短板五大任务，加快培育新动能，加快释放新活力，推动“十三五”强势开局。

2016年主要预期目标是：地区生产总值增长8%以上，一般公共预算收入增长11%，全社会固定资产投资增长15%，规模以上工业增加值增长6%左右，服务业增加值增长13%左右，社会消费品零售总额增长10%左右，出口总值增长5%，城乡居民人均可支配收入分别增长8%以上和8.5%，单位生产总值能耗和用水量、主要污染物减排、空气质量改善绩效指标完成省下达目标任务。在实际工作中，我们将向着更高的目标努力，使经济社会发展得更好更快一些。

2016年重点抓好以下九个方面的工作。

(一)全力推进稳健增长，促进经济又好又快发展。强化实体经济和重大项目支撑，发挥消费和出口拉动作用，围绕有效需求扩大投资，构建多点发力、多元支撑的增长格局。

大力发展实体经济。推动实体经济大发展，对廊坊有着特殊重要的意义。当前，实体经济面临需求弱、效益低、融资难等很多困难，在这种情况下，我们必须进一步增强发展实体经济的紧迫感和责任感，进一步把发展实体经济摆上经济发展全局的重要位置，着力推进工业强市和服务业兴市，突出发展战略性新兴产业和现代服务业，推动资源要素配置向实体经济倾斜，不断强化实体经济对经济发展的支撑作用。开展帮扶企业集中行动。紧紧围绕实体经济企业实施政企面对面帮扶，解决企业融资、用地、技术等现实问题，推动个转企、小升规，新增规上工业企业100家以上、限上商贸企业80家以上。开展降本增效专项行动。全面落实国家和省降低企业成本的一系列政策，制定实施降低企业成本总体方案，优化政府服务，降低企业交易、财务、物流、人工等各项成本，增强企业活力和竞争力。开展融资扶持行动。建立政银企多层次、多形式的常态沟通机制，鼓励发行债券、上市等直接融资，上市后备企业动态保持100家，新增挂牌上市企业20家。开展营商环境优化行动。鼓励大众创业、万众创新，培育更多市场主体;推进信用信息公开监督和信用产品市场化，提升社会信用水平;保护企业合法权益，开展企业家培训、考察活动，提升企业家素质，壮大廊商团队。

加大项目投资力度。实施项目建设“十百千”工程，申列省级重点计划新开工产业项目10个以上，实施建设市级重点产业项目100个，谋划建设亿元以上项目1000个。紧盯国家“7+4”重大工程包、国家专项建设资金和省配套资金，谋划建设一批基础设施和重大产业项目。推广政府和社会资本合作(PPP)模式，引进保险资金支持重大项目建设。

挖掘消费和出口增长潜力。开展改善消费品供给专项行动。全面落实国家和省扩大消费的政策措施，加快释放消费潜力。落实带薪休假，鼓励错峰休假、弹性作息，促进旅游消费;加快互联网宽带和无线网络建设，提高城乡物流配送能力，培育消费热点和消费市场;扩大消费信贷，培育消费金融市场，积极发展健康、医疗、教育、文化等各类消费。发挥出口对增长的促进作用。落实省外贸稳增长十条政策和市培育外贸竞争新优势若干措施，清理规范进出口环节收费，增加出口直放企业数量，支持企业参加各类展会、拓展市场，推动廊坊电子信息出口基地、霸州钢木家具基地等特色产品基地建设，提高机电、高新技术、优质农产品等出口规模。

(二)全力推进协同发展，加快同城融合发展步伐。认真落实“三个精准”要求，扎实推进重点领域率先突破，协同发展力争走在全省前列。

编制“三区一地”规划。做好与京津各项规划对接，围绕“三区一地”发展定位，逐项编制专项规划和实施方案，进一步明确目标任务、工作重点、具体举措。各县(市、区)也要按照自身在京津冀协同发展中的定位，编制相应规划，构建市县相互衔接、相互支撑的规划体系。

精准打造承接平台。谋划和争取在我市建设集中承载地，精准承接行政职能、科研培训、 公共服务等非首都功能转移。沿重要交通干线和轨道交通站点，以县城、重点镇和开发区为载体，谋划建设一批特色鲜明、功能完善、职住合一、承载力强的“微中心”。

抓好合作园区共建和重大项目建设。扎实推进北京新机场建设，切实做好征地拆迁等各项工作，确保按时高质量交付建设用地。积极跟踪国家临空经济区规划编制，加快推进我市相关总体规划和专项规划编制工作。加快建设北京亦庄·永清高新区，突出抓好项目引进和运营模式创新，组建管理机构，积极建设协同发展示范区;力促中关村固安园和永清国际服装城建设取得积极进展;积极推进大兴固安电子商务产业园、中关村(大兴)生物医药产业基地固安合作园、京冀通航产业园、霸州智能装备产业园等合作园区实质性运作。推进高精密数控机床、中核研发中心等已签约项目落地开工，加快服务机器人、清华大学OLED等已开工重大项目建设。

全力推进交通互联互通。积极做好京唐、京霸、廊涿、白沟支线和城际铁路联络线的启动建设工作，推动平谷线项目建设。加快北京新机场外围综合交通项目建设。全力争取北京城市轨道交通延伸到廊坊，积极引进北京公交进主城区。加快密涿高速公路建设，力促唐廊、津石高速公路早日开工，102国道南移项目完成前期工作，争取105国道与北京达成对接协议。加快燕郊潮白河大桥建设。

(三)全力推进转型发展，着力提升产业层次水平。按照产业政策要准的要求，坚持“加减乘除”并举，以战略性新兴产业和现代服务业为重点，全面构筑“5+3”现代产业格局。

推动新兴产业集群发展。实施新兴产业增比进位工程，着力增强电子信息、高端装备制造、新材料、生物医药等战略性新兴产业的聚集度和支撑力。加快发展以大数据为重点的电子信息产业集群。以建设廊坊云服务基地为目标，做好廊坊大数据产业发展规划和大数据产业园区规划，推动信息资源平台建设，启动工业企业云和医疗健康云建设，支持润泽科技、华为云中心、联通华北基地、新奥大数据中心等龙头企业做大做强，力促京东云计算中心、浪潮云计算中心、银联卡北方总部、京津冀量子通信、国际能源智慧谷等新项目落地开工。依托北华航天学院，实施互联网+天基信息应用工程，发展空间信息产业，建设航天遥感产品民用化示范市。继续举办大数据产业周，培育大数据众创空间和孵化转化基地。加快发展高端装备制造产业集群。制定推进“中国制造2025”实施意见，重点发展以精雕数控、华锻数控等为重点的精密机床制造，以三河京冀通航产业园、香河通航产业基地、文安长鑫航天精密组件等为重点的通航设备制造，以固安无人机、卫星导航港、香河机器人等为重点的智能制造，以中集空港专用装备、永清鼎泰智能装备等为重点的专用装备制造，以柏惠维康医疗机器人系统等为重点的医疗设备制造。加快发展新材料产业集群。大力推进中安信碳纤维、中建材薄膜太阳能电池、固安石墨烯传感器等项目建设。加快发展生物医药产业集群。积极推进固安肽谷生命科学园、原子高科同位素药物、盛唐生物科技等项目建设，力促生物医药产业链条式发展。加快发展军民融合产业集群。举办军民融合展洽会，打造省级军民融合产业示范基地。

推动现代服务业做大做强。把现代服务业作为稳增长、调结构的重要支撑，制定出台支持服务业发展政策，提升服务业对经济增长的贡献率。优先发展生产性服务业。加快发展智慧物流产业，加快西城金融商贸产业园、永清国际商贸中心、西联建材交易中心、胜记仓等项目建设;推进快递业发展，谋划建设国家一级快递枢纽节点，物流业增加值增长15%以上。加快发展金融产业，推动域外机构来廊建立分支机构，聚集金融总部结算、研发、销售等后台资源，支持民营企业发起成立民营银行，金融业增加值达到150亿元。加快发展会展业，编制发展规划，扩大开发区会展中心规模，积极承接北京会展资源，全力打造会展城市。加快发展服务外包产业，以数据处理、软件研发等为重点，聚集一批服务外包企业，争列国家服务外包示范市。积极发展生活性服务业。加快发展文创产业，不断壮大影视传媒、印刷出版、演艺娱乐、动漫游戏等产业，推动“梦廊坊”文化产业园、大城红木文化产业园、国家数字印刷产业园等文化园区建设，推进微软游戏创新中心、中信国安好莱坞影视基地、运河文化创意产业带等项目建设，文化产业营业收入增长12%以上。加快发展特色旅游业，召开旅游业发展大会，以乡村旅游为核心，整合特色资源，谋划一批采摘、观光、体验项目，举办一批推介会、博览会、节事活动，建设一批乡村旅游品牌和精品线，发展一批旅游小镇和乡村游示范村，实现旅游收入200亿元以上。加快发展健康养老产业，以建设全国养老服务业综合改革试点城市为契机，加快燕达健康城、鲁能生态度假区等龙头项目建设，打造全国智能化养老实验基地。大力培育新兴服务业态。加快发展电商产业，积极推进京东集团电商产业集群项目，做大366网上商城、齐天大圣网、富连网等本土电商平台，实施“千企在线”行动计划。依托廊坊出口加工区建设跨境电商园区。加快发展设计产业，举办中国设计节，通过设计创意催生新产业、新业态。

推动传统产业上档升级。坚定不移去产能，坚决完成省下达压减过剩产能任务，大力推动产能输出，积极处置“僵尸企业”。落实好省制造业升级专项行动，制定食品加工、木材加工及家具制造、钢铁压延及金属制品加工、绝热节能材料等传统产业转型升级方案，制定互联网+传统产业路线图，促进产业整合与跨界融合。落实省千项新产品开发和千项名牌产品培育工程，提升产品质量，打造知名品牌。启动新一轮重大技术改造升级工程，争列省技改项目60项，扶持“机器换人”重点项目20项，工业技改投资增速继续走在全省前列。

推动现代农业提质增效。大力发展现代农业园区。重点抓好省级现代农业园区建设，推进一二三产融合、产加销游一体化发展。新增省级现代农业园区4个、市级8个。大力发展农村电商。加快建设县域特色产业电商交易平台，6月底前各县(市、区)实现农村电商服务体系全覆盖，建设永清国家级电子商务进农村综合示范基地。大力推进农业产业化。围绕服务京津市场，打造高效、优质、生态、品牌农业。大力发展林下经济。推进农业产业化经营，引进建设100个农业产业化重点项目，扶持发展100家农产品加工企业，推动20家成长型龙头企业做大做强，农业产业化经营率达到68.5%。

(四)全力推进科技创新，切实增强创新发展能力。深入实施科技领引战略，着力壮大创新主体、提升创新平台、聚集创新人才，努力打造创新之城、科技之城。

培育创新主体。深入落实省高新技术产业三年倍增和科技型中小企业成长计划，推动科技型企业裂变式增长。新增科技型中小企业1000家、科技小巨人企业60家、高新技术企业25家，规上高新技术产业增加值增长13%。每个县(市、区)力争建立一个产业技术研究院或技术创新联盟，支持企业建立各类研发机构，新增市级以上各类企业研发机构80家。鼓励社会力量加大研发投入，全社会研发投入占GDP比重达到1.15%。

提升创新平台。加快建设孵化器。提升中小企业创新创业园、百世金谷、兴远高科等现有孵化器孵化能力，推进华夏“太库”、宏泰“泰智会”等孵化器建设，每个省级以上开发区至少建设一个创新创业园、一家孵化器，新增孵化面积30万平方米，争列省级孵化器2家。加快培育众创空间。做大做强中信国安创客、华航e创空间、星火创客等一批众创空间，每个县(市、区)至少打造一个高标准众创空间，支持有条件的县(市、区)建设创客新城。

强化协同创新。抓好石保廊全面创新改革试验和京南科技成果转化试验区建设，提升清华大学中试孵化基地等一批协同创新平台水平，推进中关村软件园廊坊创新中心等合作项目，加快中国(河北)博士后成果转化基地和全国智能机器人创新联盟京津冀总部建设，推进京廊科技资质互认。

优化创新环境。发展科技金融。逐步壮大现有创投基金规模，新增一批创投基金，强化财政资金撬动作用，构建以政府资本为引导、社会资本为主体的科技投融资体系。强化人才支撑。落实省科技人才“双百双千”工程，引进一批掌握重大核心技术的创新团队，培养一批技术能手和创新创业人才，支持科技人才创业，新建院士工作站2家。完善政策和服务。用足用好现有扶持政策，研究提出新的政策措施，加强政策宣传，建立科技服务、科技金融、知识产权保护等一体化服务支撑体系。

(五)全力推进生态建设，确保生态环境持续好转。把生态放在最优先的位置，坚持科学防控、精准治污，以更有力的举措，牢牢守住生态底线。

打好大气污染治理攻坚战。强力开展大气污染治理、环境执法监管和“两法一条例”百日宣传三项集中行动。加快燃煤治理和清洁能源替代，提标改造集中供热锅炉，推广煤改气、煤改电，城中村全面实施燃煤清洁化替代，实施农村清洁能源开发利用工程，大力推广环保炉具和洁净型煤。启动钢铁等重点行业脱硫、脱硝、除尘设施提标改造，对重点排放企业实施在线监测;开展VOC深度治理，有效降低臭氧浓度。全力控制扬尘，严管建筑工地和渣土运输，在主城区实行环卫“以克论净”，切实抓好城中村环境卫生整治。强化加油站和油品监管，切实加大对餐饮油烟、露天烧烤、烟花爆竹燃放、秸秆和垃圾焚烧管控力度。加强与京津联防联控，强化监测预警和信息共享，做好重污染天气应急响应。PM2.5浓度下降9%以上。

打好水环境治理攻坚战。全面贯彻水污染防治国“十条”和省“五十条”，研究制定我市治理水污染工作方案。开展重点河流污染治理攻坚行动。制定重点河流水污染防治实施方案和达标方案，强化河段长负责制，确保达到国家和省考核要求。加快污水处理厂及配套管网建设。主城区铁路北、万庄污水处理厂及配套管网力争投入运营，建设市区西部和龙河以南地区污水管网，确保龙河实现截污纳管;县城污水处理设施达到一级A排放标准。实施集中式水源地安全防护行动。严格保护水源地，推进地下水压采，南水北调配套工程全面运行，争取引调生态水常态化，扎实推进北三县引调水前期工作。

打好生态修复攻坚战。持续实施造林增绿。启动“两年大提升，创建森林城”活动，大力开展春季造林提升行动，实施重要交节点绿化、廊道绿化、连片造林等重点工程，全市铁路、国省干道、主要河道以及“精品线”绿化实现闭合，全年造林18万亩。深入实施生态修复工程。全力推进南三县湿地连片开发，努力建成东联天津、西接白洋淀的京南森林湿地生态走廊;抓好香河国家湿地公园试点，推动文安赵王新河省级湿地公园、胜芳湿地公园、龙河湿地建设，恢复湿地2万亩以上。继续实施永定河郊野公园项目，积极推动水利风景区建设和三河采矿区生态修复，实施永定河泛区安次安全区和临空安全区工程建设。推动北三县与平谷、蓟县联合申报京津冀国家生态文明先行示范区。制定土壤污染防治行动计划。开展土壤污染专项调查，落实分区保护，开展有针对性的修复试点。全面落实省对工业园区循环化改造任务。

打好环境执法攻坚战。严格落实生态环境督办问责制，开展生态环境责任审计，以督察问责促整改落实。深入开展环保执法专项行动，完善网格化监管，依法严厉打击环境违法行为。

(六)全力推进改革开放，切实破除体制机制障碍。坚持改革政策要实，积极推进重要领域和关键环节改革，强力推动改革落地。

推进两大改革试点。扎实开展全国中小城市综合改革试点，进一步完善实施方案，落实相关改革事项，探索创新规划引导、公共服务等一系列体制机制，通过三年试点，为承接北京非首都功能和产业转移做出示范。扎实推进石保廊全面创新改革试验，积极开展科技、管理、品牌、组织、商业模式全面创新，建设一批示范园区，打造一批产业创新平台，实施一批重大科技成果转化项目，为创新发展提供示范。

深化重点领域改革。大力简政放权。以“放管服”为重点，进一步增强放权的针对性、提高监管的有效性、强化服务的便利性。衔接落实好国家和省取消下放审批事项，编制市县行政许可通用目录和公共服务事项目录清单，进一步简化审批手续、规范审批程序，加强电子政务网络平台建设。深化商事制度改革。探索实行“多证合一”，试行电子营业执照，推进工商登记全程电子化。抓好社会领域改革。积极推进机关事业单位养老保险制度改革、户籍制度改革、医药卫生体制改革，6月底前完成党政机关公车改革。做好农村综合改革。加快市县农村产权交易中心建设，农村土地确权登记颁证完成全部耕地面积的80%，全面完成供销社综合改革试点任务。加快财税体制改革。开展国地税征管体制改革，高质量完成“营改增”工作，推进结构性减税和普遍性降费，积极落实京津转移产业税收分享机制。支持商业银行设立科技支行，扩大“科保贷”规模。加快用地体制机制创新。实行土地数量与强度双控，制定节约集约用地政策，强化土地供应后管理，坚决依法清理非法占地，大力盘活闲置用地，多渠道解决土地占补平衡。

提升开放发展水平。完善园区体制机制。制定开发区改革发展实施意见，召开开发区工作大会，建立省级以上开发区动态管理、末位淘汰和责任追究机制，力促园区由多向强转变。推动园区整合，探索国家级园区与域内园区合作共建、招商外包、品牌共享、利益分享等合作机制，建设一批特色区中园、国别园、科技创新孵化园。探索推动“腾笼换鸟”的具体举措，加快低质低效企业有序退出园区，为优质项目腾出空间。支持企业“走出去”。加快跨境电子商务示范企业和外贸公共服务企业建设，发展新兴服务贸易模式，鼓励优势企业开拓海外市场。加大招商引资力度。全力实施招商引资集中行动，完善常态化招商机制，实施精准化招商，着力提高招商实效性。

(七)全力推进新型城镇化，提升城乡统筹发展水平。着眼补齐发展短板，着力壮大城市经济和县域经济，努力打造产城教融合、承载力强的高端城镇平台，完成棚户区改造1.1万套，城镇化率达到55.6%。

做大做强城市经济。强化规划引领。以生态优先、绿色发展理念为指导，坚持服从服务大局、科学规划，进一步修编完善全域空间布局和城镇总体规划。加快编制海绵城市、地下管廊、综合交通、主城区停车场等系列专项规划。完善城市功能。优化城市路网，谋划实施外环路西南环线、永兴路南延、金光道东延等道路延伸和金源道高架桥建设，开展道路改造整修，完善南城路网。实施疏堵保畅集中行动，完善智能信号系统，加快城市公交专用道建设，新增新能源公交车100部，下大力解决主干道和学校周边拥堵问题。实施景观绿化工程，加快推进丹凤公园建设，开展游园提升、步行街风貌改造、外环路景观廊道建设，高标准规划环城水系改造工程。加快热电联产项目建设，力争冬季投入供暖。推进主城区重点区域免费WiFi覆盖。建设市区“5公里充电圈”。实施精细化管理，加强智慧城市建设，努力提升城市治理水平。壮大城市产业。编制中心城市产业规划，统筹优化主城区经济布局，大力发展商圈经济、楼宇经济、创意经济、总部经济，打造城市经济新的增长点。

力促县域经济增比进位。按照“三个集中”的要求，全力推进县域经济和县城建设上台阶。加强县城建设。全面修订完善县(市)城乡总体规划，完成县城建设用地控详规和专项规划编制，推进“多规合一”。深化户籍制度改革，促进人口集聚，有序扩大城镇规模。开展县城基础设施建设集中行动，实施一批园林绿化、供水供热、污水垃圾处理等项目，年内所有县城实现集中供水、集中供热或清洁能源供热，污水垃圾达标处理。建设廊坊南500千伏等一批输变电工程，强化电力保障。加强产业支撑。把园区发展与县城建设统筹谋划、同步推进，加快完善城镇功能，推动产业园区向新城镇、新城区转型。制定县域产业转型升级工作方案，每个县(市、区)谋划引进一批立市立县重大项目，扶持发展一批十亿元级优势企业，培育壮大一批百亿元级基地(园区)和产业集群，推动县域经济增比进位。大城气雾剂产业园打造全国最大气雾剂研发生产基地，霸州休闲食品产业园争列国家级食品安全示范基地。

加快美丽乡村建设。全面落实“四美”要求，突出抓实产业支撑，着力发展特色农产品、特色手工业、乡村旅游等产业。全力建设美丽乡村胜芳省级重点片区，持续提升固安和北三县省级重点片区，其他县(市、区)都要规划建设一个市级精品示范片。在264个省级重点村推进“四美五改”，实施民居改造、安全饮水等12个专项行动，不断提升美丽乡村建设水平。

(八)全力推进民生改善，尽心竭力办好惠民实事。全面贯彻共享发展理念，时刻把群众冷暖记在心上、抓在手上，深入实施八项民生工程，让全市人民有更多获得感。

居民收入提升工程。落实机关事业单位工资和津补贴改革政策，推进企业职工工资合理增长，大力治理拖欠农民工工资问题。多渠道增加居民收入，鼓励农户依法流转承包土地，增加农民财产性收入。城乡居民人均可支配收入分别达到34500元和14300元，继续保持全省领先。

就业创业促进工程。落实和完善创业扶持政策，设立创业扶持资金，建立创业贷款担保基金补充机制，落实好援企稳岗、社保补贴、税费减免等扶持政策。城镇新增就业5万人，高校毕业生就业率达到90%以上，城镇登记失业率控制在4%以内。

社保扩面提标工程。落实各项基本养老保险政策，实现困难群众养老服务补贴城乡全覆盖，做好医疗保险全市统筹，健全重特大疾病救助和疾病应急救助制度，实现大病保险全覆盖。实施社区医养结合健康养老服务工程，市区建设2个医养结合型社区居家养老示范中心，市区社区居家养老服务设施覆盖率达到95%，县级达到70%。

精准帮扶工程。加大低保兜底力度，对农村有劳动能力、具备增收条件的困难群众实施精准帮扶，丧失劳动能力的贫困人口全部纳入农村低保。开展残疾人帮扶，实施残疾儿童抢救性康复救助，培训残疾人4500名，90%以上实现稳定就业创业。对贫困群众及因病致贫家庭，落实医疗救助、临时救助和重特大疾病专项救助，使困难群众求助有门、受助及时、急难有救。

教育提质工程。继续着力缓解市区中小学“大班额”问题，市一中迁建工程实现封顶，实施市十六中新建等11所城区中小学、幼儿园建设工程。促进义务教育均衡发展，改造义务教育薄弱学校240所，创建标准化学校90所，新改扩建农村幼儿园50所。积极引进京津教育资源，鼓励社会力量办学。发展高等教育、职业教育、继续教育、网络教育、特殊教育，大力提升全民素质。做好贫困学生救助，不让一个孩子因贫失学。

医疗惠民工程。推进公立医院综合改革，完善基本药物制度，健全分级诊疗体系，加快基本医保异地就医结算。强化疾病预防控制，广泛开展全民健康教育与健康促进活动，为60岁以上老年人免费健康体检。积极承接京津优质医疗资源，鼓励社会力量办医。依法实施“全面二孩”政策。

农村设施提升工程。用好农发行等政策性金融，引导各类资金投向农村基础设施，新改建农村公路200公里以上，投资2.58亿元实施农村电网、配网改造升级，加大农村通讯信息网络建设，推进光纤通讯网络进乡入村，新增公交化农村客运班线25条，实施一批小型农田水利工程、节水配套改造工程。

文体惠民工程。完成国家公共文化服务体系示范区创建，加快市县档案馆建设，谋划启动文化产业规划馆建设，加快市群艺馆和县级“两馆”建设进度，做好文物保护和历史文化传承，组织百项文化品牌活动，推进建设千个文化惠民中心，培育万户文化示范家庭。办好市第七届全民运动会、世界围棋大赛、国际马拉松赛、第什里风筝节等重大活动，广泛开展全民健身运动，农村健身设施覆盖率达75%以上，社区达90%以上。

(九)全力推进风险防控，保持社会大局和谐稳定。廊坊区位特殊、地位重要，承担着首都“护城河”的重要职责，我们必须对防风险工作保持高度的政治自觉。强化金融风险防控。进一步完善金融监管体制，及时发现处置重大金融风险及突发事件，依法严厉打击非法集资、非法证券、非法保险等金融违法行为，坚决守住不发生系统性和区域性金融风险的底线。强化安全生产风险防控。全面落实《安全生产法》，严格实行“党政同责、一岗双责、失职追责”，深入开展重点高危行业和领域专项整治，推进安全生产隐患排查体系和安全生产标准化建设，坚决遏制重特大安全事故发生。强化食药安全风险防控。加强食品药品全程监管，推进“智能食药安全”信息化和检验检测能力建设，完善全市域网格化监管体系，开展规范化市场、食品安全示范街和示范店创建活动，依法打击制售假冒伪劣商品行为。强化公共安全风险防控。完善重大决策社会稳定风险评估机制，增强突发公共事件应急处置能力，积极应对新型业态、网络社会等带来的新风险，防范化解环境污染、土地征收等伴生风险，妥善做好化解过剩产能涉及职工转岗安置和再就业工作。加强和创新社会治理，在全省率先创新群众工作机制，市县两级群众工作中心和乡镇(村街)群众工作站(室)全部建成投用;开展信访案件化解行动，实现积案息诉息访。积极开展“平安廊坊”建设活动，提高军警民联防联治水平，深入开展“打黑除恶灭霸扫痞”专项行动，营造安定和谐的社会氛围。

加强国防动员和国防后备力量建设，做好拥军工作，支持工会、共青团、妇联等人民团体发挥桥梁纽带作用，做好新闻出版广电、民族宗教、外事侨务、人民防空、气象地震、防灾减灾、史志档案、援疆援藏等工作，促进各项事业协调发展。

四、全面加强政府自身建设

新形势新任务对政府工作提出了新要求，我们将以开展“政策落实年”、“素质提升年”活动为契机，振奋精神、锤炼作风，强化执行力、提高公信力，扎实开创政府工作新局面。

坚持解放思想，持续提升创新破难能力。自觉强化看齐意识，向党中央看齐，向党的理论和路线方针政策看齐，认真学习习近平总书记系列重要讲话精神和省委、省政府重大决策部署，用新理论新理念武装头脑。对照八破八立要求，对标先进、调高标尺，跳出廊坊看廊坊，重点解决好作风不实、落实不力等问题，坚决破除制约发展的思想障碍，以思想和能力水平的提升，推动各项工作上台阶。

坚持转变职能，持续提升服务发展能力。把“放管服”作为转变职能、自我革命的核心，在营商环境优化、公共服务供给上狠下功夫，坚决向不作为、乱作为、吃拿卡要等行为亮剑，坚决清理各类无谓证明和繁琐手续，精简办事程序，缩短办事时限。加强电子政务建设，推进全程网上审批，让群众少跑路、少受累，让市场主体切实感受到方便和快捷。

坚持依法治市，持续提升依法行政能力。始终把依法行政作为基本遵循，带头尊法、学法、守法、用法，把法治思维和法治方式贯穿于政府工作全过程，法定职责坚决作为、法无授权坚决不为。强化依法决策、依法施政，提高政府依法行政水平，强化权力监督，自觉接受人大法律监督和政协民主监督，加强审计监督和社会监督。严格规范行政执法行为，全面推进政务公开，打造“法治政府”、“阳光政府”。

坚持改进作风，持续提升推进落实能力。严格执行中央“八项规定”，认真践行“三严三实”，坚决纠正“四风”顽疾，集中整治怠政懒政不良风气。始终坚持问题导向，眼睛向下看，身子往下沉，劲头往下使。健全工作责任制度、激励机制和督查问责机制，推进作风建设常态化、长效化。以等不起、慢不得的危机感、紧迫感，夙兴夜寐、激情工作、事争一流，真正干成一批大事实事，以优良作风开创廊坊发展新局面。

坚持干净干事，持续提升廉洁持政能力。把纪律和规矩挺在前面，严守《中国共产党廉洁自律准则》和《中国共产党纪律处分条例》，严格执行廉洁自律各项规定。大力推进反腐倡廉，落实“两个责任”和“一岗双责”。强力正风肃纪，严查群众身边的腐败问题，做到有腐必反、有贪必肃，努力实现干部清正、政府清廉、政治清明。

各位代表，奋斗成就梦想，实干铸就辉煌。让我们紧密团结在以习近平同志为总书记的党中央周围，在省委、省政府和市委坚强领导下，牢记使命、勇于担当，扎实苦干、奋发作为，全力推进绿色、高端、率先、和谐发展，努力走出一条生态优先、绿色发展新路，为全面建成小康社会、谱写中华民族伟大复兴中国梦的廊坊篇章而努力奋斗!
